# Expression and Localization of Opioid Receptors in Male Germ Cells and the Implication for Mouse Spermatogenesis

**DOI:** 10.1371/journal.pone.0152162

**Published:** 2016-03-31

**Authors:** Haizea Estomba, Iraia Muñoa-Hoyos, Marta Gianzo, Itziar Urizar-Arenaza, Luis Casis, Jon Irazusta, Nerea Subirán

**Affiliations:** Department of Physiology, Faculty of Medicine and Dentistry, University of the Basque Country, Leioa, Bizkaia, Spain; University Hospital of Münster, GERMANY

## Abstract

The presence of endogenous opioid peptides in different testicular cell types has been extensively characterized and provides evidence for the participation of the opioid system in the regulation of testicular function. However, the exact role of the opioid system during the spermatogenesis has remained controversial since the presence of the mu-, delta- and kappa-opioid receptors in spermatogenic cells was yet to be demonstrated. Through a combination of quantitative real-time PCR, immunofluorescence, immunohistochemistry and flow cytometry approaches, we report for the first time the presence of active mu-, delta- and kappa-opioid receptors in mouse male germ cells. They show an exposition time-dependent response to opioid agonist, hence suggesting their active involvement in spermatogenesis. Our results contribute to understanding the role of the opioid receptors in the spermatogenesis and could help to develop new strategies to employ the opioid system as a biochemical tool for the diagnosis and treatment of male infertility.

## Introduction

Over 80 million people worldwide experience infertility and over one-third of infertility cases are due to male factors. Male infertility often reflects faults in spermatogenesis but little is known about the underlying causes, mostly because mechanisms and pathways involved in spermatogenesis remain unknown.

The most well-known physiological effect associated with endogenous opioid peptides (EOPs) is their efficacy in pain reduction or analgesia, although their effect on a variety of other physiological functions has become apparent in recent years [1]. In particular, evidence of the widespread presence of EOPs and receptors in different organs and tissues of the male reproductive system indicates that EOPs likely participate in the regulation of male reproductive function [2]. EOPs are involved in cell communication and exert their action through G-protein-coupled opioid receptors. There are three principal types of opioid receptors: the mu-opioid (MOR), delta-opioid (DOR) and kappa-opioid (KOR) receptors [3]. Later, the orphanin 1 (ORL1) receptor (also known as the nociceptin receptor) was discovered and found to have high homology with opioid receptors [4].Our group described the presence of MOR, DOR and KOR and the other components of the opioid system in human sperm cells which seem to be functionally involved in control of human sperm motility [5–9]. However, the presence of MOR, DOR and KOR in male germ cells and their roles during spermatogenesis remain unknown.

Spermatogenesis is a highly coordinated developmental process characterized by mitotic, meiotic and haploid differentiation phases. Spermatogenesis is initiated in the basal compartment of the seminiferous epithelium by spermatogonial stem cells that proliferate and differentiate into type A1 spermatogonia. Type A1 spermatogonia undergo a series of synchronized mitotic divisions, giving rise to type B spermatogonia, which enter the meiotic phase of spermatogenesis as primary spermatocytes [10]. Meiosis is characterized by two consecutive cell divisions, following a single DNA duplication, and by genetic exchange (crossing-over) between homologous chromosomes, which results in four round haploid spermatids [11]. EOPs are present in different cells of the male gonads and likely intervene in the mechanisms that regulate spermatogenesis. Opioid protein precursors are expressed differentially in somatic and germ cells of the testes, indicating that EOPs may regulate testicular function locally by *de novo* synthesis [2]. Moreover, Leydig cells also synthesize EOPs in the mouse and these opioid peptides may be involved in control of spermatogenesis by inhibiting the function of Sertoli cells in a paracrine. Specifically, EPOs inhibit the production of Androgen Binding Protein (ABP) stimulated by FSH in Sertoli cells [12]. ABP is responsible for testosterone transport into the lumen of the seminiferous tubule regulating intratubular testosterone levels[13]. Fabbri et al. [14] reported the presence of the three types of opioid receptors—MOR, DOR and KOR—in the rat testis using binding studies. However, subsequent higher resolution localization studies found that these receptors were exclusively expressed by Sertoli cells manner. Only ORL1 have been described in spermatogenic cells [15]. The presence of these receptors in mature spermatozoa [5] suggests that opioid receptors may be expressed at some point during spermatogenesis since mature spermatozoa are transcriptionally and translationally inactive cells. Because the effect of opioid receptors on spermatogenic cells remains unknown, the aim of this study was to characterize the expression and distribution of the three types of opioid receptors in male germ cells and analyze their role during spermatogenesis.

## Materials and Methods

### Isolation of stage-specific segments of seminiferous epithelium and mouse testicular cells

Experiments were conducted in compliance with the Spanish legislation for the use of animals for experimental purposes and approved by Ethics Committee for Animal Experimentation (CEEA) of the University of the Basque Country.

Two-month-old Swiss male mice (n = 120) were euthanized through cervical dislocation. The obtained testes were decapsulated in phosphate buffer saline (PBS) to obtain the seminiferous tubules. We used the transillumination-assisted microdissection technique to obtain the stage-specific segments of seminiferous tubules [16]. We used different stages of the seminiferous epithelium dissected out under transillumination to analyze the gene expression of opioid receptors in male germ cells and isolated total testicular cells for immunofluorescence analysis, functional experiments, and FACS analyses. To isolate total cells, testis were decapsulated and dissociated in 2 mg mL^–1^ of collagenase for 15 min, followed by 0.25% trypsin/1 mM EDTA digestion for 10 min at 37°C in KREBS medium supplemented with 10% foetal bovine serum (FBS). Finally, the cells were filtered with nylon of 41-μm pore size nylon (Millipore, Germany)

### Cell culture and and Opioid receptor agonist treatments

Isolated testicular cells were resuspended and cultured on Roswell Park Memorial Institute 1640 medium containing 10% knockout serum replacement (KSR) from Gibco (Invitrogen, California USA), penicillin/streptomycin (50 U/mL) and 200 mM L-glutamine. Except for KSR, the reagents were purchased from Sigma-Aldrich (St. Louis, MO). The spermatogenic cells were placed on culture dishes for 24h. Subsequently, we treated with selective opioid receptors agonists for short- (1 h) and long-term exposure (24 h). We used the MOR selective agonist morphine (10^−5^ M, Alcaliber S.A., Madrid, Spain), DOR selective agonist [d-Pen2,d-Pen5]-enkephalin (DPDPE) (10^−6^ M, Sigma-Aldrich) and KOR selective agonist U-50488 (10^−6^ M, Sigma-Aldrich).

### qRT-PCR

Total RNA was extracted with Nucleo Spin RNAII Kit (Macherey-Nagel, Germany) according to the manufacturer’s instructions. Concentrations of RNA were determined by measuring absorbance at 260 nm.Purity was assessed by the 260/280 nm absorbance ratio. For synthesis of first-strand cDNA, we mixed the RNA with random hexamers, dNTP and SuperScript II Reverse Transcriptase (Invitrogen) in a total volume of 20 μL.The cDNA samples were added to a 20-μL reaction mixture containing primers (Table 1) and Power SYBR Green PCR Master Mix (Applied Biosystems, California, USA). PCR reaction profile was:40 cycles of 15 s at 95°C (denaturation) and 1 min at 60°C (annealing and extension) (Abiprism 7000 Sequence Detection System). Quantification of gene expression was calculated from the standard curves of each gene using the same RNA sample per triplicates. Data were normalized with beta-actin (*Actb*) as a housekeeping gene measured in the same biological sample. In order to identify the most stable reference genes, we analyzed changes in expression of TATA binding protein (*Tbp*), glyceraldehyde 3-phosphate dehydrogenase (*Gapdh*) and beta-actin (*Actb*) gene expression by geNorm software. According to the calculated geNorm M value (M) *Actb* was the most stable reference gene in our system. Moreover, *Actb* was used as a housekeeping gene in testicular cells [17].

**Table 1:** Primers.

Gene	Primers (forward/reverse)	Biological significance
*mMOR*	(f) TGTCGGAGAACTGAGAGCAA	Mu-opioid Receptor
	(r) CCTGAACTGTGGAAGGAAGC	
*mDOR*	(f) TGTAAAGAGGGCTGGGAATG	Delta-opioid Receptor
	(r) TTGGTTTGAGGGTTGGTTTT	
*mKOR*	(f) TGACTTGGGAAGGGAGGTC	Kappa-opioid receptor
	(r) AGCACTGGGAGAGCAGGTA	
*mITGA6*	(f) GCTTCCTCGTTTGGCTATGA	Marker for spermatogonia
	(r) AATCGGCTTCACATTACTCCA	
*mSYCP1*	(f) GCTTTTGGGAGAGGTTGAGA	Marker for spermatocytes
	(r) CGCTGATGACTGTTCTTGCT	
*mSYCP3*	(f) ATCTGGGAAGCCACCTTTG	Marker for spermatocytes
	(r) AGCCTTTTCATCAGCAACATC	
*mACR*	(r) GAGTGAAGAAGGACGGGTTG	Marker for spermatids
	(f) CAGGAGCAAGAAGAGCAGGA	
*mACTB*	(f) GGGCTATGCTCTCCCTCAC	Beta-actin: Housekeeping gene
	(r) CACGCACGATTTCCCTCT	

### Immunofluorescence

For the immunofluorescence staining, isolated testicular cells were fixed with 4% paraformaldehyde in PBS and permeabilized with Triton X-100 0.5% in PBS. Cells were blocked in 10% fetal bovine serum (FBS) and then primary antibodies were added (Table 2). Negative controls were performed with the rabbit immunoglobulin fraction, omitting the primary antibody before secondary antibody, andnuclei were stained with Hoechst 33342 at 0.5 μg mL^–1^.Slides were assembled with Fluoromount G (Molecular Probes) and samples were examined by confocal microscopy (Olympus Fluoview FV500). To elucidate the levels of opioid receptors present in different spermatogenic cells, we have quantified the **“**Corrected total cell fluorescence” (CTCF) per area measured by ImageJ software. We measured the green fluorescence of at least 100 cells. To avoid changes influenced by the area of spermatogenic cell, we have expressed the CTCF per area of the cells using the following equation: CTCF/ microm^2^ = [Integrated density–(Area of selected cell × Mean fluorescence of background readings)] / Area of selected cell.

**Table 2:** Antibodies.

Antibody	Dilution	Reference
Rabbit anti-MOR	1:1000	Millipore (AB5511)
Rabbit anti-DOR	1:1000	Millipore (AB1580)
Rabbit anti-KOR	1:1000	Millipore (ABN456)
Mouse anti-ITGA6	1:1000	Abcam (ab75737)
Goat anti-SYCP1	1:700	Santa Cruz (sc20837)
Mouse anti-SYCP3	1:1000	Santa Cruz (sc74956)
Goat anti-ACR	1:500	Santa Cruz (sc46284)
Anti-Rabbit IgG: Alexa Fluor 488	1: 1000	Life Technologies (A21206)
Anti-Mouse IgG: Alexa Fluor 647	1:1000	Life Technologies (A11008)
Anti-Goat IgG: Alexa Fluor 633	1:500	Life Technologies (A21082)

### Immunohistochemistry

Swiss male mice were anaesthetized with 3-aminobenzoic acid ethyl ester (MS-222), perfused with Bouin’s solution. Testes were removed and immersed in the same fixative for 12 h. The samples were embedded in paraffin and 5 μm-thick sections were obtained. Briefly, antigen retrieval was carried out in citrate buffer (10 mM, pH = 6) for 30 minutes at 98°C in microwave oven. Endogenous peroxidase activity was blocked by incubating the slides in 3% hydrogen peroxide in PBS for 30 min. Samples were permeabilized with Triton X-100 0.1% in PBS (TBS) for 10 min. Cells were blocked in 10% normal goat serum and bovine serum albumin (1mg/ml) for 30 min and the primary antibodies, Rabbit anti-Mu Opioid Receptor (1:100, Millipore), Rabbit anti-Delta Opioid Receptor (1:100, Millipore) and Rabbit anti-Kappa Opioid Receptor antibodies (1:200, Millipore), were applied overnight at 4°C in the same blocking medium. A subsequent reaction was performed with biotinylated secondary antibody. Complex of avidin-biotin peroxidase was formed for 1h and positive cells were visualized using 3,3-diaminobenzidine tetrahydrochloride plus (DAB+) as a chromogen. Counterstaining was performed with hematoxylin. Negative controls were performed omitting the primary antibody before secondary antibody addition. Digital slides were acquired with a MIRAX SCAN (Zeiss) and images captured with the Cell Software.

### Cell cycle analysis by flow cytometry

Isolated testicular cells were fixed in 70% ethanol at 4°C for 1 h and resuspended and incubated in staining solution (PBS with 0.2 mg mL^–1^ RNase A and 0.02 mg mL^–1^ propidium iodide) at 4°C for 1 h. We used lymphocytes as a control to identify the 2c DNA-containing cell population because these cells are non-mitotic cells. Fluorescence data from 100,000 events were collected in the FL2 sensor (GalliosTM, Becton Dickinson, San Jose, CA, USA). To quantify the effects of different treatments on haploid cells we measured the integrated area of the peaks corresponding to 1c DNA-containing cells. Histograms were analyzed using Summit v4.3 software. All experiments were repeated six times using biological replicates from six different animals, with three technical replicates from each isolation experiment.

### Statistics

Statistical analysis was performed using Student's t-test and analysis of variance (ANOVA) followed by a post hoc analysis using the Bonferroni test. These procedures were carried out with GraphPad PRISM (version 5.0) program. Differences were considered significant for p< 0.05.

## Results and Discussion

Numerous studies have demonstrated the presence of EOPs in different testicular cell types [12–15,18–20], providing evidence that the opioid system is important in the regulation of testicular function. Binding studies have shown that the three types of opioid receptors–MOR, DOR and KOR–are exclusively expressed by Sertoli cells [14]. The present study demonstrates for the first time that active MOR, DOR and KOR are present in male germ cells and may regulate the coordination and orchestration of cellular proliferation and differentiation events that take place at several points along the spermatogenesis.

### Expression and localization of opioid receptors in male germ cells

**Fig 1:**
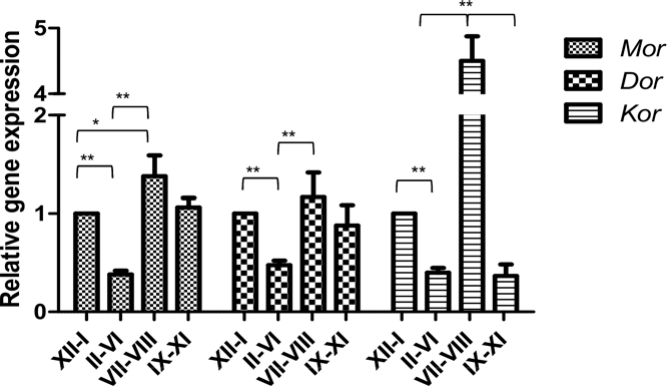
Expression of opioid receptors in mouse spermatogenic cells. Developmental gene expression of *Mor*, *Dor* and *Kor* at stages XII–I, II–VI, VII–VII and IX–XI of seminiferous tubules according to qRT-PCR. Data were normalized with beta-actin (*Actb*) as a housekeeping gene and presented as relative expression related to XII–I stage. Mean ± SEM of six experiments. *P<0.05; **P<0.01.

The expression of opioid receptors is developmentally regulated during spermatogenesis and is consistent with spermatogenesis being characterized by highly specialized mechanisms of gene expression that operate at transcriptional and post-transcriptional levels [18–19]. To clarify the presence of opioid receptors in germ cells, we first examined the gene expression of receptors in different stages of the seminiferous epithelium using qRT-PCR in Swiss adult mouse testis. qRT-PCR analysis showed that *Mor*, *Dor* and *Kor* were expressed at different stages (Fig 1) with a similar expression pattern as detected at the 12-stage sperm development in mouse [21]. At stages II-VI, expression of the three receptors steeply dropped compared respected to XII–I (*P* < 0.01). During stages VII–VIII, however, the respective expressions of *Mor* (3.5-fold), *Dor* (3-fold) and *Kor* (11-fold) (*P* < 0.01) increased compared to stages II–VI.

**Fig 2:**
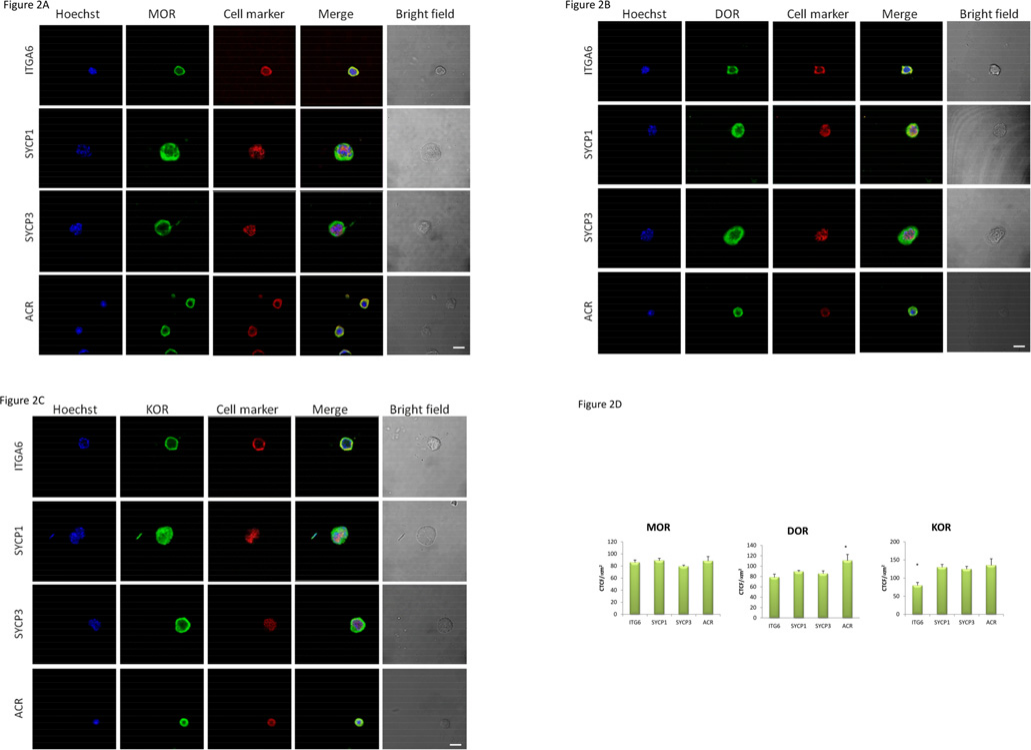
Localization of opioid receptors in mouse spermatogenic cells. Co-immunoflorescence analysis of MOR (A), DOR (B) and KOR (C) with male germ cell markers; with integrin alpha-6 (ITGA6), synaptonemal complex proteins SYCP1 and SYCP3, and acrosin (ACR) as cell markers of spermatogonia, spermatocytes and spermatids, respectively. Nuclei were stained with Hoechst 33342. Negative controls were treated with preimmune rabbit serum and secondary antibody alone. Representative photomicrographs are shown; n = 5. Scale bar: 10 μm. Intensity fluorescence for MOR, DOR and KOR was measured using Corrected total cell fluorescence per area (D). CTCF/microm^2^ = [Integrated density–(Area of selected cell × Mean fluorescence of background readings)] / Area of selected cell. Only green fluorescence was measured for at least 100 cells. *P<0.05.

In agreement with previous data [14], testicular cells expressed the opioid receptors. However, different stages of the seminiferous epithelium contained distinct groups of spermatogonia, spermatocytes and spermatids at various points of development. To determine which male germ cell receptors were expressed, we examined their expression and localization by double immunofluorescence using specific germ cell markers. We used integrin alpha-6 (ITGA6), synaptonemal complex proteins SYCP1 and SYCP3, and acrosin (ACR) as cell markers of spermatogonia [22], spermatocytes [23] and spermatids [24], respectively. The double indirect immunofluorescence revealed that all male germ cells expressed the three opioid receptors (Fig 2). There was strong staining for the receptors in peripheral regions of all cells, confirming their membrane localization. The three opioid receptors were present in spermatogonia–we observed MOR- (Fig 2A), DOR- (Fig 2B) and KOR- (Fig 2C) positive cells that co-expressed ITGA6 in the peripheral regions (ITGA6 is a membrane protein) [22]. Cell morphology also confirmed the nature of the cell. Moreover, spermatocytes also expressed opioid receptors. There was strong immunostaining of MOR, DOR and KOR in SYCP1- and SYCP3-positive cells that correspond to spermatocytes (Fig 2A, 2B and 2C). Immunoreactivity of SYCP1 and SYCP3 was detected in the nucleus of cells [25]. Spermatids also expressed opioid receptors. Moreover, the presence of MOR, DOR and KOR in haploid cells was confirmed by ACR. In round spermatids, ACR-positive cells were co-stained with MOR (Fig 2A), DOR (Fig 2B) and KOR (Fig 2C). ACR was localized in the cytoplasm of round spermatids as we expected [24]. We also detected a strong immunostaining of opioid receptors in mouse spermatozoa (S1 Fig). According to CTCF/microm^2^ measurements, MOR expression did not change during spermatogenesis (Fig 2D). We did not observe any differences in the MOR fluorescence intensity between ITGA6, SYCP1, SYPC3 and ACR positive cells. DOR fluorescence intensity, however, increased in ACR positive (p<0.05) cells compared to ITGA6, SYCP1 and SYCP3-positive cells respected. There was also an increase in KOR fluorescence intensity in SYCP1, SYCP3 and ACR positive cells compared to ITGA6-positive cells (p<0.05).

**Fig 3:**
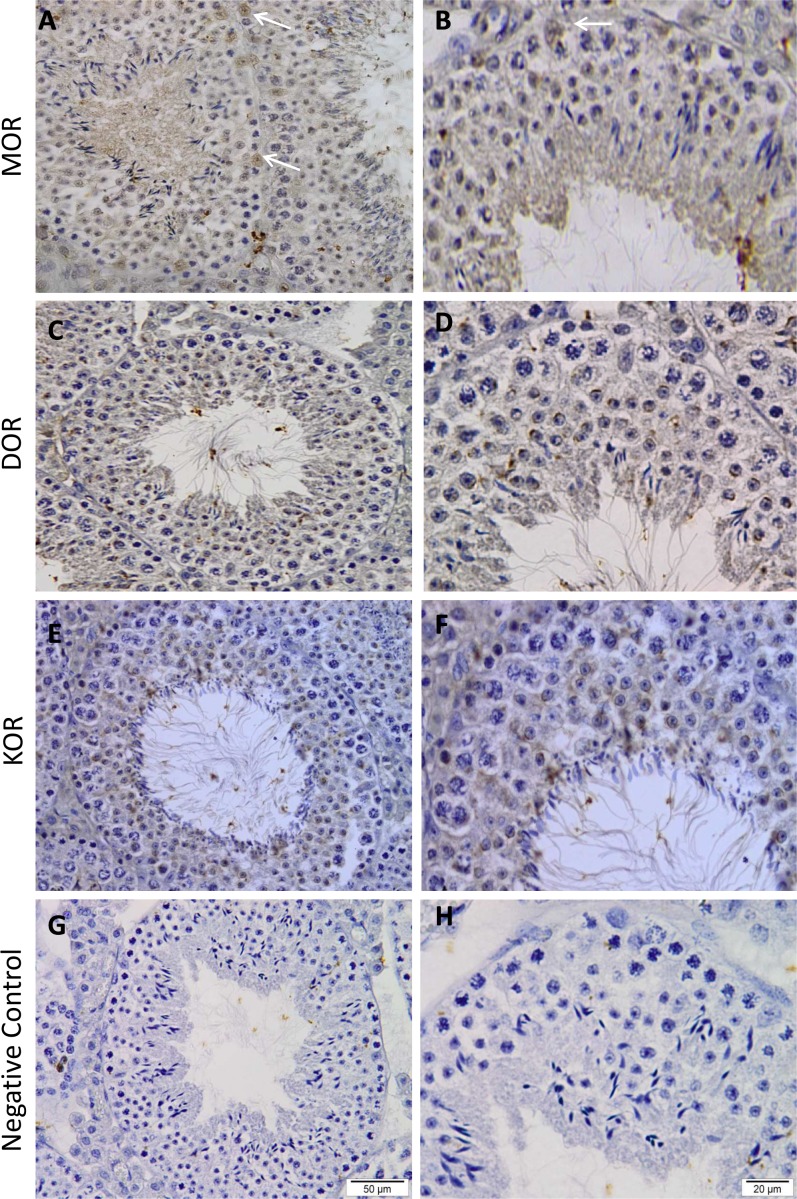
Histological localization of opioid receptors in mouse testis. Immunohistochemical expression of MOR (A and B), DOR (C and D) and KOR (E and F) in representative mouse testis. Opioid receptors are widely expressed along mouse testis. White arrows represent Sertoli cells represent Sertoli cells. Counterstaining was performed with hematoxylin. Negative controls were performed omitting the primary antibody before secondary antibody addition (G and H). n = 3. Scale bar: 100 μm.

To verify the presence of opioid receptors in spermatogenic cells we carried out immunohistochemistry analysis to identify cells in their histological context (Fig 3). Immunoreactivity of the three opioid receptors had been detected widely expressed inside seminiferous tubules confirming the presence of these receptors in male germ cells where these cells are placed. Specifically, we observed a stronger immunoreactivity of DOR over the acrosome region in spermatids and sperm cells (Fig 2B, Fig 3 and S1 Fig). We have also observed a strong immunoreactivity of MOR in Sertoli cells (Fig 3) as has been described before [14].

Thus, immunofluorescence and immunohistochemistry analyses indicated the presence of MOR, DOR and KOR in different cell types at different spermatogenic cells. These findings suggest a central role of the opioid system in regulation of spermatogenesis.

Spermatogenesis is a series of processes, involving the proliferation of spermatogonia, the meiosis and the differentiation of developing germ cells (spermatids) into sperm. To investigate whether the opioid receptors were related to spermatogenesis, we analyzed the effect of opioid receptor agonists on the spermatogenic cell cycle and on expression of sperm germ-cell markers for different time exposures. Because spermatogenesis is controlled by paracrine and autocrine processes that take place between different cells types present in the testicles, we decided to culture all the testicular cells. We have demonstrated for the first time that MOR, DOR and KOR were present in mice male germ cells and can be involved in the spermatogenesis regulation.

A proliferative effect of opioid receptors was previously reported [1,3]. Indeed, it has been suggested that opioid receptors can stimulate some proliferative pathways such as neurogenesis or adrenal regeneration [26–30] and inhibit the growth of immature adrenals or macrophage proliferation in the peripheral nervous system [28, 29]. Thus, we described for the first time the role of opioid receptors in sperm cell differentiation. Our data suggest that opioid receptors were functionally active, with the response being time-exposure dependent.

### The effect of MOR-agonist morphine on spermatogenesis

**Fig 4:**
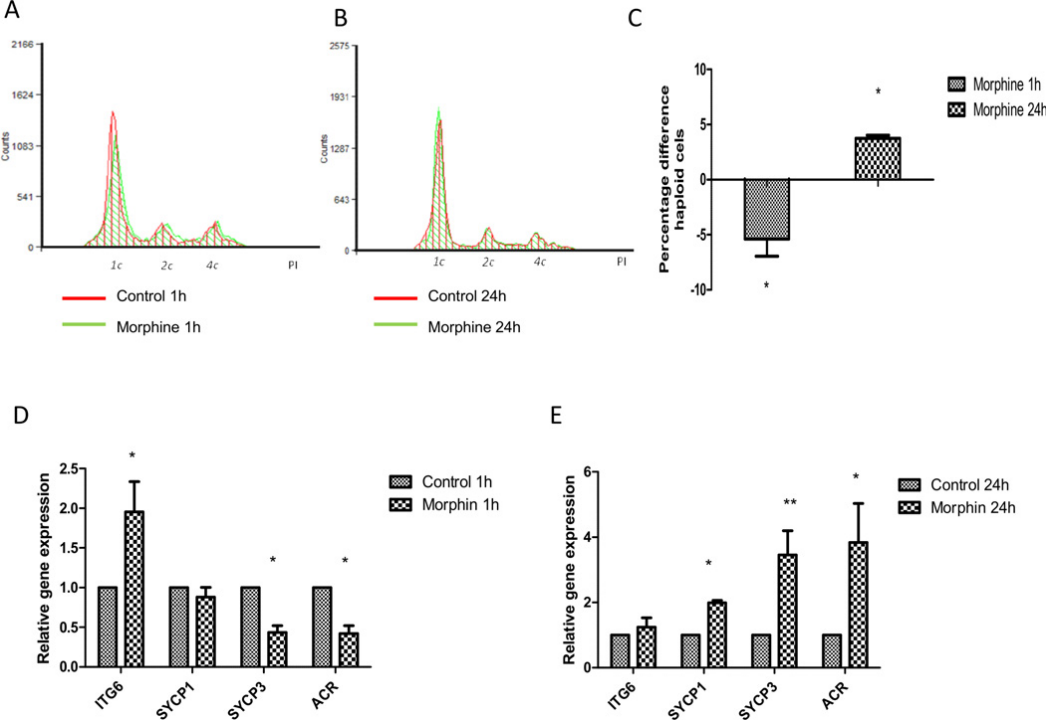
Effect of selective MOR-agonist morphine on spermatogenesis. Flow cytometry histogram of spermatogenesis cell cycle measured by propidium iodide in control (green) and morphine-treated samples (red: 10^−5^ M) for short- (A) and long-term exposure (B). Representative plot from six experiments. Percentage difference between treatment and control of the integrated area of the haploid cell population for the different times presented as relative expression mean ± SEM of six experiments (C). Relative gene expression measured by qRT-PCR of germ cell markers *Itga6*, *Sycp1*, *Sycp3* and *Acr* for short- (D) and long-term exposure (E) with morphine. Data were normalized with beta-actin (*Actb*) as a housekeeping gene and presented as relative expression related to control. Mean ± SEM of six experiments. *P<0.05 vs. control; **P<0.01 vs. control.

To determine the role of opioid receptors in mouse spermatogenesis we studied the changes in the spermatogenic cycle by flow cytometry. Flow cytometry analysis showed that acute morphine treatment caused a decrease in the 1c DNA-contained cells population and an increase in the 2c DNA-contained cells population (Fig 4A, S2 Fig). However, chronic morphine treatment increased haploid population (Fig 4B). We observed an increase in the 1c DNA-contained cells in treated samples than in control ones together with a down-regulation of 2c and 4c DNA-contained cell population (Fig 4B, S2 Fig). Due to the presence of somatic cells, we measured only the percentage of haploid cells to evaluate the direct effect of opioid receptors on spermatogenesis since changes in the number of 2c and 4c cells could be influenced. Measuring the integrated area corresponding to peaks of haploid cells showed that the percentage of 1c DNA-containing cells decreased by 5% in treated samples (*P* < 0.05) compared to controls (Fig 4C). However, chronic morphine treatment increased the haploid cell population by approximately 5% (*P* < 0.05) (Fig 4C).

To determine whether MOR was involved in different phases of spermatogenesis, we analyzed the expression of *Itga6*, the synaptonemal complex proteins *Sycp1* and *Sycp3*, and *Acr* by qRT-PCR. The relative expression of *Itga6* increased (*P* < 0.05) after 1 h of morphine treatment (Fig 4D); however, *Sycp3* mRNA levels decreased (*P* < 0.05) with acute morphine treatment and there was no effect on *Sycp1* levels. Finally, we also observed a decrease in *Acr* expression (*P* < 0.05), the spermatid cell marker, consistent with acute morphine treatment causing a decrease in haploid cells. To understand the increase in haploid cells induced by morphine for long-term exposure; we also analyzed the expression of spermatogenic cell markers after 24 h of incubation. The chronic opioid treatment led to an increase not only in *Acr* mRNA levels (*P* < 0.05), but also in *Sycp1* (*P* < 0.05) and *Sycp3* (*P* < 0.01) levels (Fig 4E)–confirming the increase in percentage of haploid cells measured by flow cytometry, Long-term exposure to morphine did not affect *Itga6* expression.

### The effect of DOR-agonist DPDPE on spermatogenesis

**Fig 5:**
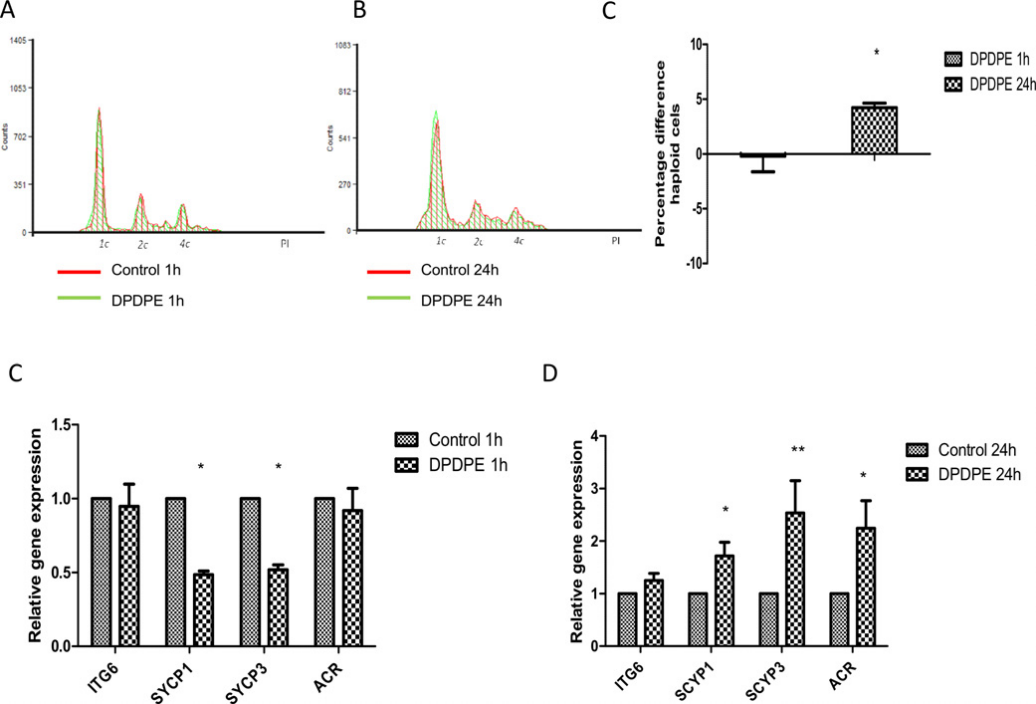
Effect of selective DOR-agonist DPDPE on spermatogenesis. Flow cytometry histogram of spermatogenesis cell cycle measured by propidium iodide in control (green) and DPDPE-treated samples (red: 10^−6^ M) for short- (A) and long-term exposure (B). Representative plot from six experiments. Percentage difference between treatment and control of the integrated area of the haploid cell population for the different times presented as relative expression mean ± SEM of six experiments (C). Relative gene expression measured by qRT-PCR of germ cell markers *Itga6*, *Sycp1*, *Sycp3* and *Acr* for short- (D) and long-term exposure (E) with DPDPE. Data were normalized with beta-actin (Actb) as a housekeeping gene and presented as relative expression related to control. Mean ± SEM of six experiments. *P<0.05 vs. control; **P<0.01 vs. control.

To determine the role of DOR in mouse spermatogenesis, we studied the changes induced by DOR-agonist DPDPE in the spermatogenic cycle using flow cytometry for short- (1 h) and long-term exposure (24 h). DPDPE did not cause any effect on the 1c nor 2c DNA-containing cell population after 1 h of treatment and led to a significant increase in 4c DNA-containing cell population (Fig 5A, S3 Fig). For long-term exposure, however, the haploid as well as the diploid cell population increased after treatment while DPDPE caused a decrease in the 4c DNA-containing cell population. (Fig 5B, S3 Fig). To evaluate the direct effect of DOR on spermatogenesis we measured the integrated area corresponding to peaks of haploid cells (Fig 5C, S3 Fig). We have measured an approximately 5% increase in haploid cells with long-term exposure (*P* < 0.05), suggesting that DOR receptor was also active in the spermatogenic cycle.

To determine whether DOR was involved in different phases of spermatogenesis, we analyzed changes on the expression of cell markers after DPDP treatment. Our results showed that DOR was mainly involved in the meiosis phase. The relative expression of *Sycp1* and *Sycp3* (*P* < 0.05) decreased after 1 h of DPDPE treatment (Fig 5D) while, in regard of spermatogonia marker *Itga6* and haploid cell marker *Acr*, we did not observed differences in the mRNA levels for short-term exposure (Fig 5A and 5C). On the other hand, there was an increase in the expression of *Sycp1* (*P* < 0.05), *Sycp3* (*P* < 0.01) and *Acr* mRNA levels (*P*< 0.05) after 24 h of DPDPE incubation (Fig 5E). These data confirmed the increase in the haploid cell population induced by DPDPE for long-term exposure suggesting that DOR had an effect on spermatogenesis for long-term exposure.

### The effect of KOR-agonist U50488 on spermatogenesis

**Fig 6:**
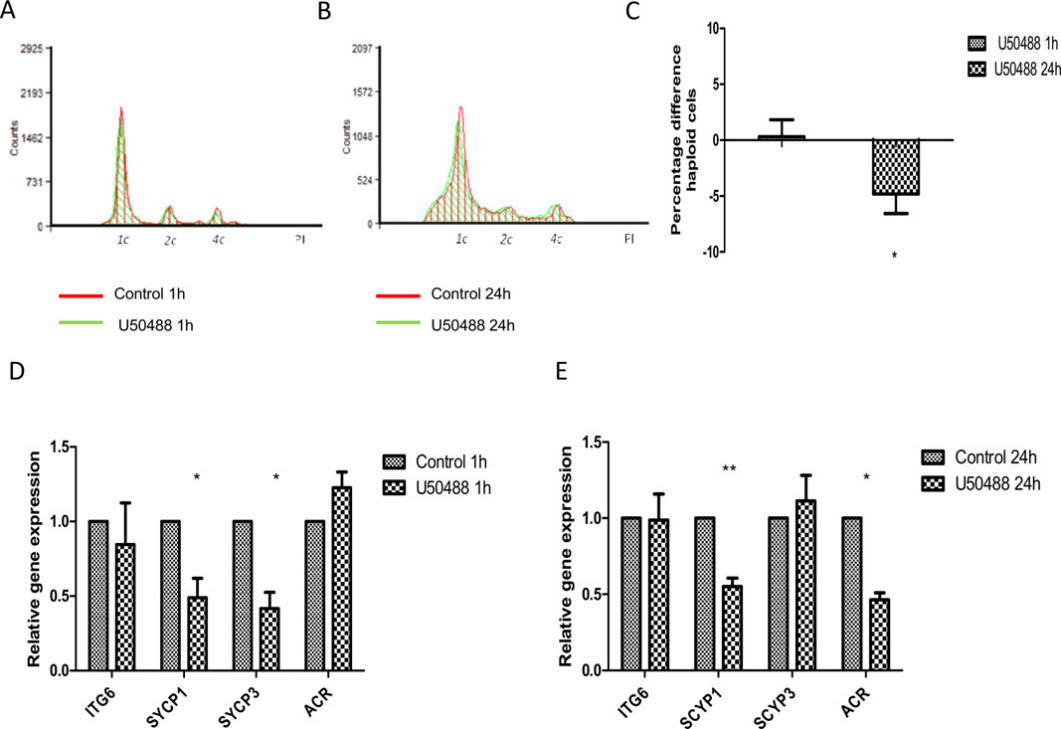
Effect of selective KOR-agonist U50488 on spermatogenesis. Flow cytometry histogram of spermatogenesis cell cycle measured by propidium iodide in control (green) and U50488-treated samples (red: 10^−6^ M) for short- (A) and long-term exposure (B). Representative plot from six experiments. Percentage difference between treatment and control of the integrated area of the haploid cell population for the different times presented as relative expression mean ± SEM of six experiments (C). Relative gene expression measured by qRT-PCR of germ cell markers *Itga6*, *Sycp1*, *Sycp3* and *Acr* for short- (D) and long-term exposure (E) with U50488. Data were normalized with beta-actin (*ACTB*) as a housekeeping gene and presented as relative expression related to control. Mean ± SEM of six experiments. *P<0.05 vs. control; **P<0.01 vs. control.

To determine the role of KOR in mouse spermatogenesis, we carried out the same experimental design using U50488 as KOR the agonist for short- (1 h) and long-term exposure (24 h). For short-term exposure, there were no differences in the 1c, 2c nor 4c DNA-containing cell populations (Fig 6A, S4 Fig). However, U50488 caused a down-regulation of haploid cells together with an up regulation of the 2c- and 4c- DNA-containing cell populations for long-term exposure (Fig 6B, S4 Fig). Measuring the integrated area corresponding to peaks of haploid cells showed an approximately 5% decrease in haploid cell numbers after long-term exposure (Fig 6C, S4 Fig; *P* < 0.05) suggesting that KOR was also active in spermatogenic cells similar to the other receptors. Expression of cell markers showed that KOR was also involved in the meiosis phase. Analysis by qRT-PCR showed a decrease in *Sycp1* and *Sycp3* expression (*P* < 0.05) after treatment with U50488 for 1 h, but there were no differences in mRNA levels of haploid cell marker *Acr* (Fig 6D). With long-term exposure, however, U50488 led to a decrease in *Sycp1* (*P* < 0.01) and *Acr* (*P* < 0.05) expression, confirming the flow cytometry results (Fig 6E) whereas the expression of *Itga6* remained unchanged.

In summary, opioid receptors acted as modulators of spermatogenesis since MOR, DOR and KOR knockout mice are not completely infertile [31]. For short-term exposure, the decrease of haploid cells observed after 1 h suggests that MOR selective agonist inhibited the spermatogenic processes and the decrease of *Acr* expression after agonist treatment also confirmed the inhibitory effect of this receptor on spermatogenesis. However, we observed the opposite effect for long-term exposure. Not only MOR but also DOR selective agonists promoted spermatid marker *Acr* expression and the haploid cell differentiation, suggesting an activating role of both receptors on spermatogenesis. Similarly to other receptors [7,17], the activation of compensatory mechanisms can explain the opposite effect of MOR and DOR with long exposure times. However, further analyses will be needed in this regard. KOR was also involved in cell proliferation but, in this case, the selective agonist, U50488, produced only a decrease in the haploid cell population and in *Acr* expression with long-term exposure. This suggests that KOR had an inhibitory effect on spermatogenesis as it has been described for this receptor in other processes [30].

Our results also suggest that the three opioid receptors can be involved in meiosis of mice spermatogenesis by modifying the gene expression of the synaptonemal complex proteins SYCP1 and SYCP3. During the first meiotic division, alignment, synapsis and meiotic recombination are the most crucial events ensuring proper chromosome segregation. Synapsis is mediated by the synaptonemal complex, a meiosis-specific structure that assembles between aligned homologous chromosomes, where SYCP1 and SYCP3 proteins act as a structural framework for recruitment of further protein components [23]. MOR and DOR activation may produce defects in synapsis with short-term exposure by down-regulation of *Sycp3* expression, and *Sycp1* and *Sycp3* expression, respectively. SYCP1 and SYCP3 knockout mice are infertile [32]. On the one hand, SYCP1 knockout mice are unable to assemble synaptonemal complex and the homologous chromosomes fail to synapse [33] and, on the other hand, SYCP3 is essential for the synaptonemal complex assembly and for accurate meiotic chromosome compaction [34,35]. The activation of KOR also causes down-regulation of *Sycp1* with long-term exposure, suggesting that KOR may also inhibit synapsis.

We also observed that long-term exposure to selective MOR and DOR agonists caused an increase in *Sycp1* and *Sycp3* gene expression (Figs 4D and 5D). The up-regulation of both, *Sycp1* and *Sycp3*, is also related to defects in homologous recombination and appears to be associated with the induction of mitotic catastrophe and the generation of endopolyploid tumour cells [36,37]. Thus, our results suggest that the three opioid receptors were negatively involved in meiosis of mice spermatogenesis by modifying *Sycp1 and Sycp3* expression, since defective synapses and meiotic recombination have severe consequences for meiosis progression that can lead to infertility or aneuploidy in mammals [23].

## Conclusions

The present study demonstrated for the first time that male germ cells expressed active MOR, DOR and KOR in mouse. The main conclusions are following:

1. Active mu-opioid, delta-opioid and kappa-opioid receptors were present in male germ cells in mouse testes.
2. The opioid receptors mu-opioid, delta-opioid and kappa-opioid are involved in the regulation of mouse spermatogenesis.
3. The three opioid receptors seemed to be involved in the regulation of male meiosis in mouse modifying the gene expression of synaptonemal complex proteins

Our results contribute to resolving several longstanding issues concerning the role of opioid receptors in spermatogenesis. These include how chronic morphine administration may reduce fertility in rats [38], how methadone administration for 3 days can produce spermatocyte chromosomal aberrations in mouse [39] and how transgenic mouse that overexpress pro-enkephalin (an endogenous opioid peptide protein precursor) in the testes have impaired fertility, morphologically abnormal testicles and low sperm motility [40]. These findings open up novel avenues of research of the opioid system as a biochemical tool for the diagnosis and treatment of male infertility.

## Supporting Information

S1 Fig Localization of opioid receptors in mouse spermatozoa. Immunoflorescence analysis of MOR, DOR and KOR with male germ cell markers. Negative controls were treated with preimmune rabbit serum and secondary antibody alone. Nuclei were stained with Hoechst 33258. Representative photomicrographs are shown; n = 5.
(JPG)

S2 Fig Effect of selective MOR-agonist morphine on spermatogenic cell cycle. Flow cytometry histogram of spermatogenesis cell cycle measured by propidium iodide in control (green) and morphine-treated samples (red: 10^−6^ M) for short- (A) and long-term exposure (B). Representative plot from six experiments. Changes on percentages of 1c, 2c and 4c DNA-containing cell populations for short- (C) and long-term exposure (D) after morphine treatment. Percentage difference between treatment and control of the integrated area of the 1c, 2c and 4c DNA-containing cell population for the different times presented as relative expression mean ± SEM of six experiments (E). *P<0.05 vs. control; **P<0.01 vs. control.
(JPG)

S3 Fig Effect of selective DOR-agonist morphine on spermatogenic cell cycle. Flow cytometry histogram of spermatogenesis cell cycle measured by propidium iodide in control (green) and DPDPE-treated samples (red: 10^−6^ M) for short- (A) and long-term exposure (B). Representative plot from six experiments. Changes on percentages of 1c, 2c and 4c DNA-containing cell populations for short- (C) and long-term exposure (D) after DPDPE treatment. Percentage difference between treatment and control of the integrated area of the 1c, 2c and 4c DNA-containing cell population for the different times presented as relative expression mean ± SEM of six experiments (E). *P<0.05 vs. control; **P<0.01 vs. control.
(JPG)

S4 Fig Effect of selective KOR-agonist morphine on spermatogenic cell cycle. Flow cytometry histogram of spermatogenesis cell cycle measured by propidium iodide in control (green) and U50488-treated samples (red: 10^−6^ M) for short- (A) and long-term exposure (B). Representative plot from six experiments. Changes on percentages of 1c, 2c and 4c DNA-containing cell populations for short- (C) and long-term exposure (D) after U50488 treatment. Percentage difference between treatment and control of the integrated area of the 1c, 2c and 4c DNA-containing cell population for the different times presented as relative expression mean ± SEM of six experiments (E). *P<0.05 vs. control; **P<0.01 vs. control.
(JPG)

## References

[1] Feng Y, He X, Yang Y, Chao D, Lazarus LH, Xia Y (2012) Current research on opioid receptor function. Curr Drug Targets. 2;13(2):230–46. 22204322 10.2174/138945012799201612 PMC3371376

[2] Subiran N, Casis L, Irazusta J. (2011) Regulation of Male Fertility by the Opioid System. Mol Med. 17(7–8):846–53. 10.2119/molmed.2010.00268 21431247 PMC3146603

[3] Gaveriáux-Ruff C, and Kieffer BL. (1999) Opioid receptors: gene structure and function Opioids in Pain Control: Basic and Clinical Aspects. Cambridge University Press.

[4] Mollereau C, et al Parmentier M, Mailleux P, Butour JL, Moisand C, Chalon P, at al. (1994) ORL1, a novel member of the opioid receptor family: cloning, functional expresion and localization. FEBS Lett. 341:33–8. 8137918 10.1016/0014-5793(94)80235-1

[5] Fernandez D, Valdivia A, Irazusta J, Ochoa C, Casis L. (2002) Peptidase activities in human semen. Peptides. 23: 461–8. 11835995 10.1016/s0196-9781(01)00622-2

[6] Irazusta J, Valdivia A, Fernández D, Agirregoitia E, Ochoa C, Casis L. (2004) Enkephalin-degrading enzymes in normal and subfertile human semen. J Androl.25(5):733–9 15292103 10.1002/j.1939-4640.2004.tb02848.x

[7] Agirregoitia E, Valdivia A, Carracedo A, Casis L, Gil J, Subiran N, et al (2006) Expression and localization of delta-, kappa-, and mu-opioid receptors in human spermatozoa and implications for sperm motility. J Clin Endocrinol Metab. 91: 4969–75. 16984994 10.1210/jc.2006-0599

[8] Subiran N, Agirregoitia E, Valdivia A, Ochoa C, Casis L, Irazusta J. (2008) Expression of enkephalin-degrading enzymes in human semen and implications for sperm motility. Fertil Steril. 89: 1571–7. 17953966 10.1016/j.fertnstert.2007.06.056

[9] Subirán N, Candenas L, Pinto FM, Cejudo-Roman A, Agirregoitia E, Irazusta J. (2012) Autocrine regulation of human sperm motility by the met-enkephalin opioid peptide. Fertil Steril. 98(3):617–625. 10.1016/j.fertnstert.2012.05.036 22749218

[10] de Rooij DG (2001) Proliferation and differentiation of spermatogonial stem cells. Reproduction. 121:347–354. 11226060 10.1530/rep.0.1210347

[11] Roeder GS (1997) Meiotic chromosomes: It takes two to tango. Genes Dev. 11:2600–2621. 9334324 10.1101/gad.11.20.2600

[12] Fabbri A, Knox G, Buczko E, Dufau ML (1988) Beta-endorphin production by the fetal Leydig cell: regulation and implications for paracrine control of Sertoli cell function. Endocrinology. 122(2):749–55 2962854 10.1210/endo-122-2-749

[13] Huang HF, Pogach LM, Nathan E, Giglio W, Seebode JJ (1991) Synergistic effects of follicle-stimulating hormone and testosterone on the maintenance of spermiogenesis in hypophysectomized rats: relationship with the androgen-binding protein status. Endocrinology 128(6):3152–61 1903701 10.1210/endo-128-6-3152

[14] Fabbri A, Tsai-Morris CH, Luna S, Fraioli F, Dufau ML(1985) Opiate receptors are present in the rat testis. Identification and localization in Sertoli cells. Endocrinology 117(6):2544–6 2998740 10.1210/endo-117-6-2544

[15] Eto K, Shiotsuki M, Abe S. (2013) Nociceptin induces Rec8 phosphorylation and meiosis in postnatal murine testes. Endocrinology. 8;154(8):2891–9. 10.1210/en.2012-1977 23720425

[16] Kotaja N, Kimmins S, Brancorsini S, Hentsch D, Vonesch JL, Davidson I, et al (2004) Preparation, isolation and characterization of stage-specific spermatogenic cells for cellular and molecular analysis. Nat Methods. 12;1(3):249–54. 16144087 10.1038/nmeth1204-249

[17] Grimaldi P, Orlando P, Di Siena S, Lolicato F, Petrosino S, Bisogno T, et al (2009) The endocannabinoid system and pivotal role of the CB2 receptor in mouse spermatogenesis. Proc Natl Acad Sci U S A. 7 7;106(27):11131–6. 10.1073/pnas.0812789106 19541620 PMC2708706

[18] Tsong SD, Phillips D, Halmi N, Liotta AS, Margioris A, Bardin CW, et al (1982) ACTH and beta-endorphin-related peptides are present in multiple sites in the reproductive tract of the male rat. Endocrinology. 110(6):2204–6 6280990 10.1210/endo-110-6-2204

[19] Fabbri A, Knox G, Buczko E, Dufau ML. (1988) Beta-endorphin production by the fetal Leydig cell: regulation and implications for paracrine control of Sertoli cell function. Endocrinology. 122(2):749–55 2962854 10.1210/endo-122-2-749

[20] Soverchia L, Mosconi G, Ruggeri B, Ballarini P, Catone G, Degl'Innocenti S, et al (2006) Proopiomelanocortin gene expression and beta-endorphin localization in the pituitary, testis, and epididymis of stallion. Mol Reprod Dev. 73(1):1–8 16177984 10.1002/mrd.20341

[21] Russell LD (1990) Histological and Histopathological Evaluation of the Testis, Clearwater, FL: Cache River Press pp. 119–161.,

[22] Phillips BT, Gassei K, Orwig KE. (2010) Spermatogonial stem cell regulation and spermatogenesis. Philos Trans R Soc Lond B Biol Sci.5 27;365(1546):1663–78 10.1098/rstb.2010.0026 20403877 PMC2871929

[23] Fraune J, Schramm S, Alsheimer M, Benavente R. (2012) The mammalian synaptonemal complex: protein components, assembly and role in meiotic recombination. Exp Cell Res. 7 15;318(12):1340–6. 10.1016/j.yexcr.2012.02.018 22394509

[24] Sato T, Katagiri K, Gohbara A, Inoue K, Ogonuki N, Ogura A, et al (2011) In vitro production of functional sperm in cultured neonatal mouse testes. Nature. 3 24;471(7339):504–7 10.1038/nature09850 21430778

[25] Qiao H, Chen JK, Reynolds A, Höög C, Paddy M, Hunter N. (2012) Interplay between synaptonemal complex, homologous recombination, and centromeres during mammalian meiosis. PLoS Genet. 6;8(6):e1002790 10.1371/journal.pgen.1002790 22761591 PMC3386176

[26] Narita M, Kuzumaki N, Miyatake M, et al (2006). Role of delta-opioid receptor function in neurogenesis and neuroprotection. J Neurochem. 97(5):1494–505. 16696856 10.1111/j.1471-4159.2006.03849.x

[27] Kim E, Clark AL, Kiss A, Hahn JW, Wesselschmidt R, Coscia CJ, et al (2006) Mu- and kappa-opioids induce the differentiation of embryonic stem cells to neural progenitors. J Biol Chem. 11 3;281(44):33749–60. 16954126 10.1074/jbc.M603862200 PMC2587057

[28] Hayward NJ, McKnight AT, Woodruff GN. (1993). Neuroprotective effect of the kappa-agonist enadoline (CI-977) in rat models of focal cerebral ischaemia. Eur J Neurosc. 5(7):961–7. 10.1111/j.1460-9568.1993.tb00947.x 8281306

[29] Malendowicz LK, Rebuffat P, Tortorella C, Nussdorfer GG, Ziolkowska A, Hochol A. (2005) Effects of met-enkephalin on cell proliferation in different models of adrenocortical-cell growth. Int J Mol Med. 15(5):841–5 15806307

[30] Aita M1, Byers MR, Chavkin C, Xu M. (2010) Trigeminal injury causes kappa opioid-dependent allodynic, glial and immune cell responses in mice. Mol Pain. 1 29;6:8 10.1186/1744-8069-6-8 20109235 PMC2826348

[31] Kieffer BL. (1999) Opioids: first lessons from knockout mice. Trends Pharmacol Sci. 1;20(1):19–26. 10101958 10.1016/s0165-6147(98)01279-6

[32] Kouznetsova A, Benavente R, Pastink A, Höög C. (2011) Meiosis in mice without a synaptonemal complex. PLoS One. 6(12):e28255 10.1371/journal.pone.0028255 22164254 PMC3229524

[33] de Vries FA, de Boer E, van den Bosch M, Baarends WM, Ooms M, Yuan L, et al (2005) Mouse Sycp1 functions in synaptonemal complex assembly, meiotic recombination, and XY body formation. Genes Dev. 6 1;19(11):1376–89. 15937223 10.1101/gad.329705 PMC1142560

[34] Yuan L, Liu JG, Zhao J, Brundell E, Daneholt B, Hoog C. (2000) The murine SCP3 gene is required for synaptonemal complex assembly, chromosome synapsis, and male fertility. Mol. Cell 5 73–83. 10678170 10.1016/s1097-2765(00)80404-9

[35] Baudat F, Imai Y, de Massy B. (2013) Meiotic recombination in mammals: localization and regulation. Nat Rev Genet. 11;14(11):794–806. 10.1038/nrg3573 24136506

[36] Kalejs M, Ivanov A, Plakhins G, Cragg MS, Emzinsh D, Illidge TM, et al (2006) Upregulation of meiosis-specific genes in lymphoma cell lines following genotoxic insult and induction of mitotic catastrophe. BMC Cancer. 1 9;6:6 16401344 10.1186/1471-2407-6-6 PMC1351196

[37] Hosoya N, Okajima M, Kinomura A, Fujii Y, Hiyama T, Sun J, Tashiro S et al (2011) Synaptonemal complex protein SYCP3 impairs mitotic recombination by interfering with BRCA2. EMBO Rep.12 23;13(1):44–51 10.1038/embor.2011.221 22116401 PMC3246250

[38] Cicero TJ, Davis LA, LaRegina MC, Meyer ER, Schlegel MS. (2002) Chronic opiate exposure in the male rat adversely affects fertility. Pharmacol Biochem Behav. 5;72(1–2):157–63. 11900783 10.1016/s0091-3057(01)00751-1

[39] Badr FM, Rabouh SA, Badr RS. (1979) On the mutagenicity of methadone hydrochloride. Induced dominant lethal mutation and spermatocyte chromosomal aberrations in treated males. Mutat Res. 11;68(3):235–49. 514304 10.1016/0165-1218(79)90155-1

[40] O'Hara BF, Donovan DM, Lindberg I, Brannock MT, Ricker DD, Moffatt CA, et al (1994) Proenkephalin transgenic mice: a short promoter confers high testis expression and reduced fertility. Mol Reprod Dev 38(3):275–84 7917279 10.1002/mrd.1080380308

