# Supplementary figures and images for: Expression and Localization of Opioid Receptors in Male Germ Cells and the Implication for Mouse Spermatogenesis
Source: PLoS One. 2016 Mar 31;11(3):e0152162. doi: 10.1371/journal.pone.0152162 (PMC4816522; doi:10.1371/journal.pone.0152162)

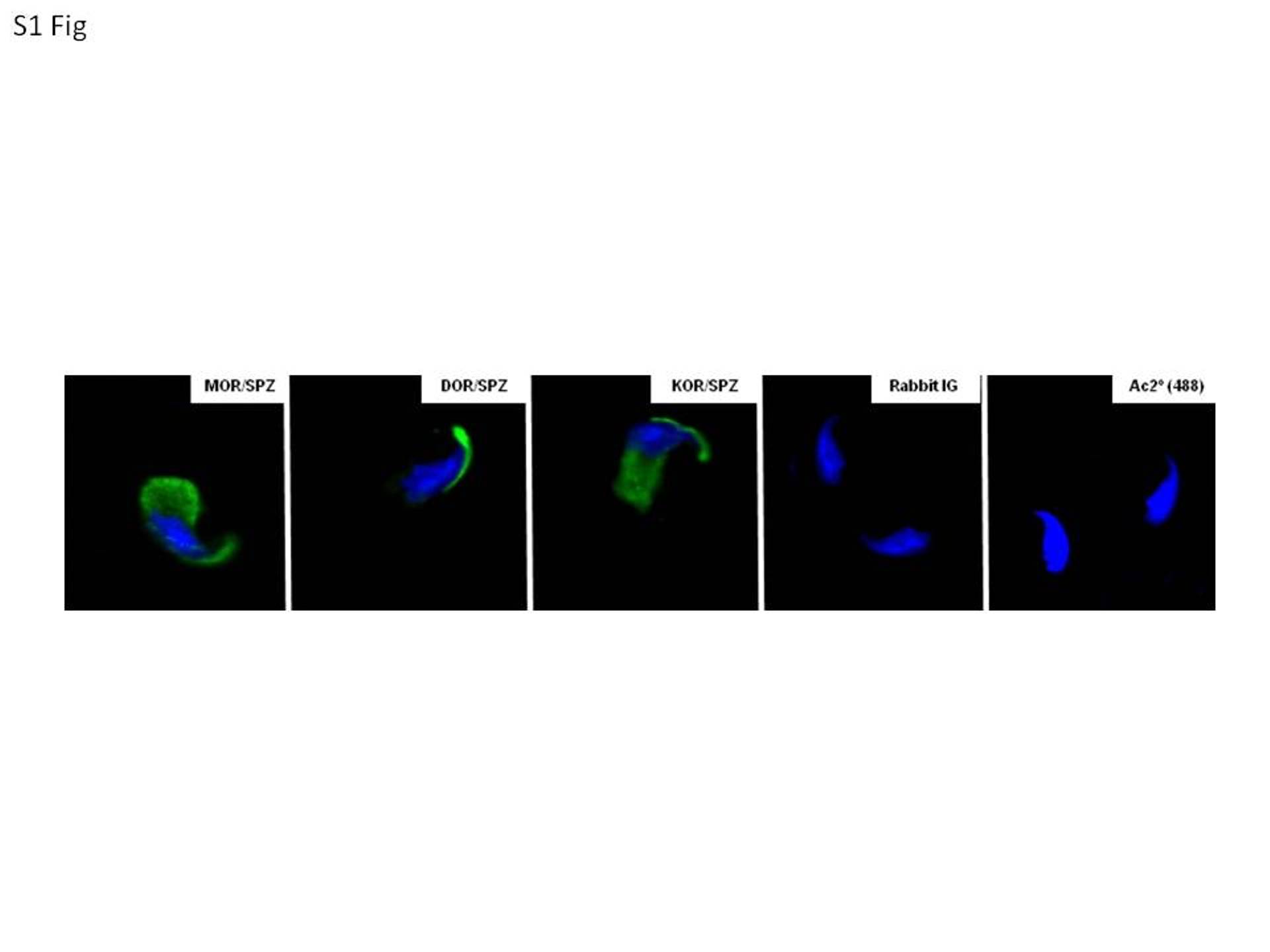

Supplement: S1 Fig — Immunoflorescence analysis of MOR, DOR and KOR with male germ cell markers. Negative controls were treated with preimmune rabbit serum and secondary antibody alone. Nuclei were stained with Hoechst 33258. Representative photomicrographs are shown; n = 5. (JPG) [file pone.0152162.s001.JPG]

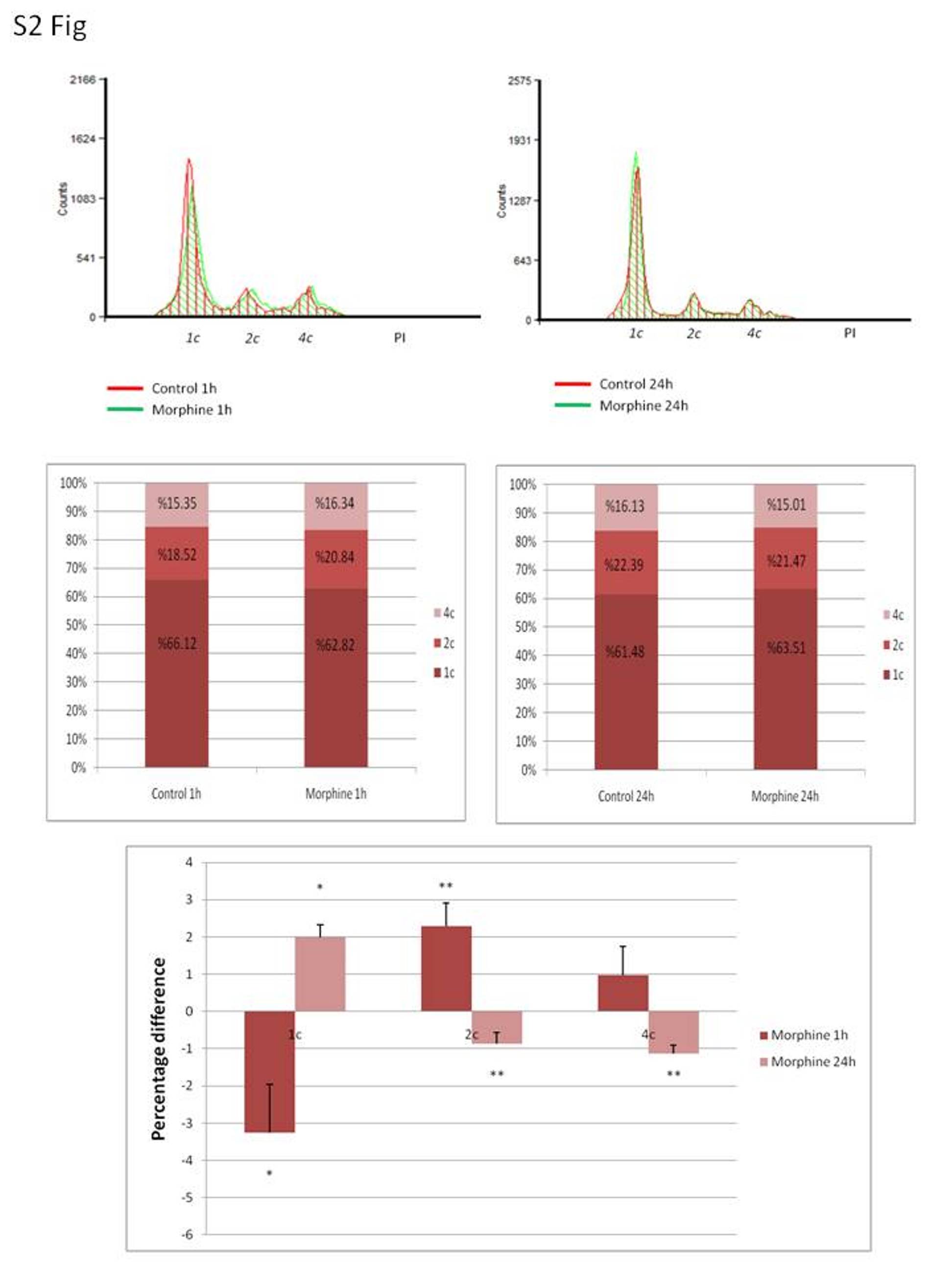

Supplement: S2 Fig — Flow cytometry histogram of spermatogenesis cell cycle measured by propidium iodide in control (green) and morphine-treated samples (red: 10−6 M) for short- (A) and long-term exposure (B). Representative plot from six experiments. Changes on percentages of 1c, 2c and 4c DNA-containing cell populations for short- (C) and long-term exposure (D) after morphine treatment. Percentage difference between treatment and control of the integrated area of the 1c, 2c and 4c DNA-containing cell population for the different times presented as relative expression mean ± SEM of six experiments (E). *P<0.05 vs. control; **P<0.01 vs. control. (JPG) [file pone.0152162.s002.JPG]

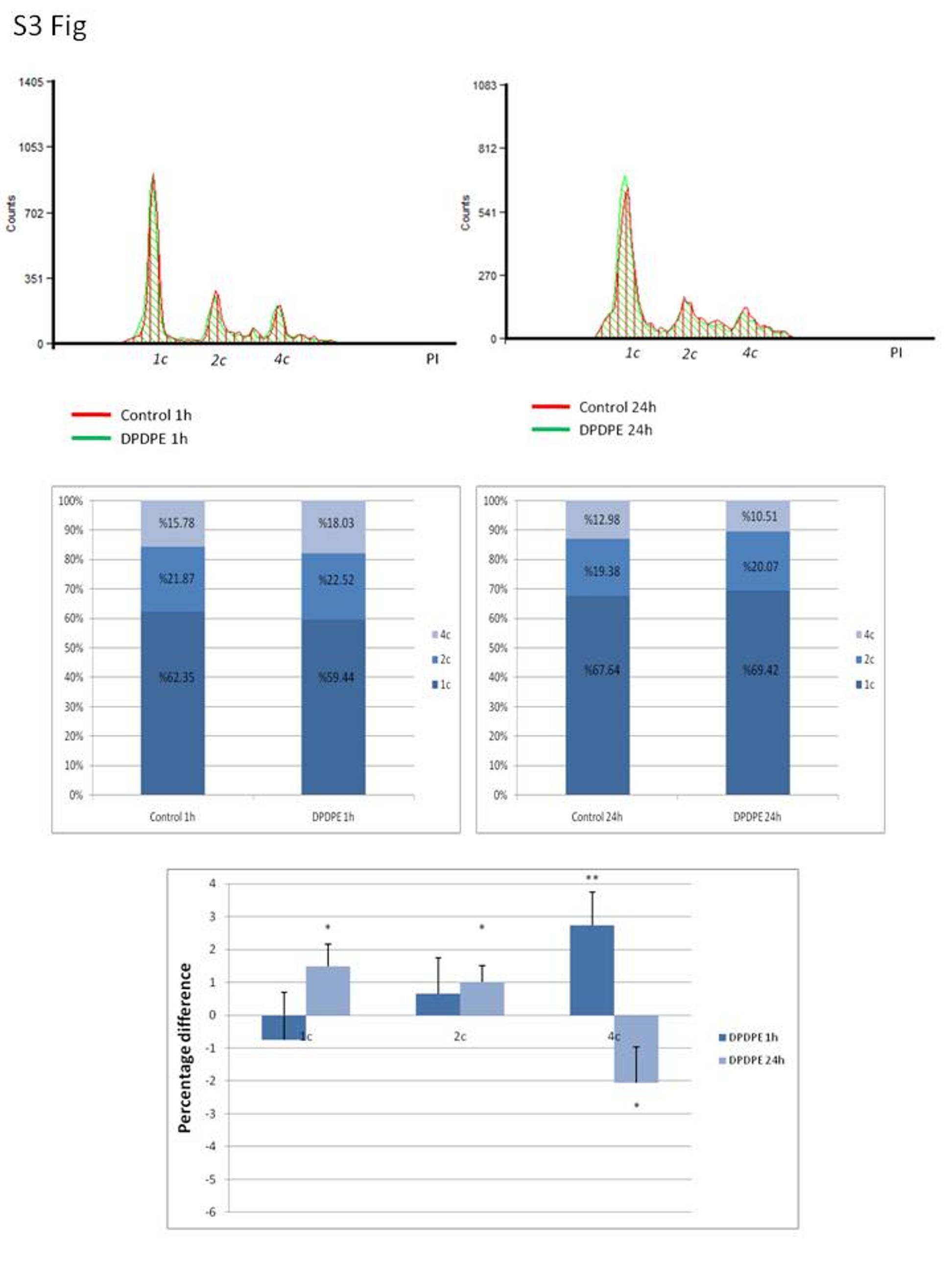

Supplement: S3 Fig — Flow cytometry histogram of spermatogenesis cell cycle measured by propidium iodide in control (green) and DPDPE-treated samples (red: 10−6 M) for short- (A) and long-term exposure (B). Representative plot from six experiments. Changes on percentages of 1c, 2c and 4c DNA-containing cell populations for short- (C) and long-term exposure (D) after DPDPE treatment. Percentage difference between treatment and control of the integrated area of the 1c, 2c and 4c DNA-containing cell population for the different times presented as relative expression mean ± SEM of six experiments (E). *P<0.05 vs. control; **P<0.01 vs. control. (JPG) [file pone.0152162.s003.JPG]

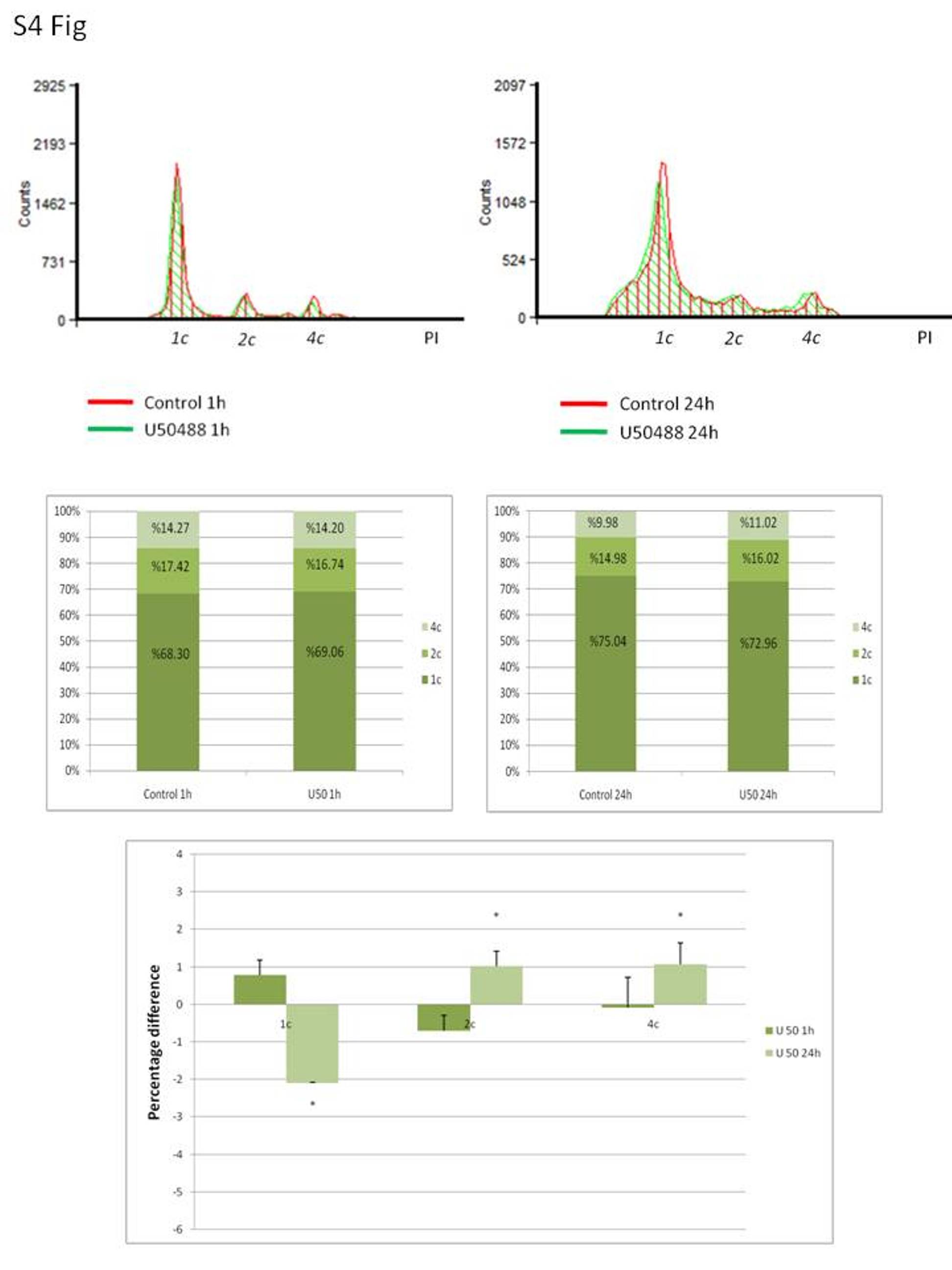

Supplement: S4 Fig — Flow cytometry histogram of spermatogenesis cell cycle measured by propidium iodide in control (green) and U50488-treated samples (red: 10−6 M) for short- (A) and long-term exposure (B). Representative plot from six experiments. Changes on percentages of 1c, 2c and 4c DNA-containing cell populations for short- (C) and long-term exposure (D) after U50488 treatment. Percentage difference between treatment and control of the integrated area of the 1c, 2c and 4c DNA-containing cell population for the different times presented as relative expression mean ± SEM of six experiments (E). *P<0.05 vs. control; **P<0.01 vs. control. (JPG) [file pone.0152162.s004.JPG]
